# Competition Between *Lemna minuta, Lemna minor*, and *Azolla filiculoides*. Growing Fast or Being Steadfast?

**DOI:** 10.3389/fchem.2018.00207

**Published:** 2018-06-14

**Authors:** Simona Paolacci, Marcel A. K. Jansen, Simon Harrison

**Affiliations:** ^1^Enterprise Center Distillery Field, School of Biological, Earth and Environmental Sciences, University College of Cork, Cork, Ireland; ^2^Environmental Research Institute, University College of Cork, Cork, Ireland

**Keywords:** Lemnaceae, Azolla, invasive species, competiton, distribution pattern

## Abstract

A substantial number of Lemnaceae are invasive outside their natural distribution area. *Lemna minuta* is considered invasive in several European countries, where it can occur in the same habitat as invasive *Azolla filiculoides* and native *Lemna minor*. In this study the presence, abundance and growth rates of all three species were monitored across 24 natural ponds and in a series of mesocosms in order to explore the importance of species invasiveness and habitat invisibility. Field monitoring showed that the distribution of the three species of macrophytes is heterogeneous in space and time. However, the data show no association of nutrient or light levels with plant distribution. Indeed, using reciprocal transplanting experiments it was demonstrated that all species are able to grow in all ponds, even ponds where the species do not naturally occur. It is concluded that distribution of *L. minor, L. minuta*, and *A. filiculoides* is not limited by the prevailing physicochemical characteristics of the ponds during the summer period. Remarkably, in these experiments *A. filiculoides* displayed the highest RGR, and exerted a negative influence on growth rates and surface cover of *L. minor* and *L. minuta*. Despite such apparent invasiveness, *A. filiculoides* was relatively rare in the study area. Rather, the species most abundant was *L. minor* which has the lowest RGR under field conditions in summer. Therefore, this study shows that the invasiveness of the species during the summer months is not necessarily reflected in the actual distribution pattern in natural ponds. In fact, alien *L. minuta* and *A. filiculoides* are under-represented in the monitored area. It is concluded that the interaction of several factors, including growth under winter-conditions and/or dispersal after disturbances, is the major determinant of the abundance and heterogeneous distribution of *L. minor, L. minuta*, and *A. filiculoides* in the study area.

## Introduction

Biological invasions have been increasing over the past 50 years (Levine and D'Antonio, 2003) and these invasions are a source of concern because of their negative effects on native species, habitats and biodiversity (McGeoch et al., 2010). Alien aquatic plants can have a negative impact on ponds, streams, rivers and wetlands. The dense growth of some alien aquatic plants can reduce flora richness and structural diversity and cause alterations in ecosystem function (Zedler and Kercher, 2004). Invasions can also have serious economic implications, particularly if they affect food production, shipping, water-extraction, fisheries, tourism, and/or recreation. Across all ecosystems, there are estimated to be more than 1,000 invasive alien species in Europe that have been shown to cause a substantial ecological or economic impact (Vilà et al., 2009).

Understanding the factors that promote the invasiveness of alien species is fundamental in order to prevent invasions and restore invaded habitats (Byers et al., 2002). The ability of plants to invade a habitat is called invasiveness while, the susceptibility of an environment to the colonization and establishment by species not currently part of the resident community, is called invasibility (Davis et al., 2005). A biological invasion depends on both the invasiveness of the alien species and the invasibility of the habitat (Alpert et al., 2000). The degree of invasibility of a habitat depends on many factors including the species richness and the strength of interactions between species (Case, 1990). Resource availability, disturbance and environmental stressors have also been demonstrated to have an impact on the invasibility of habitats (Davis et al., 2000). Among the traits that seem to be correlated with a high invasiveness of a species are a broad native distribution range (Goodwin et al., 1999), rapid population growth (Rejmánek and Richardson, 1996), ability to deal with stress and disturbance and rapid dispersal (Alpert et al., 2000). The competitive strength of an alien species, relative to native species, also impacts on the success of an alien invasion (Alpert et al., 2000).

A substantial number of Lemnaceae species do occur outside their natural distribution range, and are considered to be invasive. For example, in Sweden five different species of non-native Lemnaceae have been found (*Lemna aequinoctialis, L. minuta, L. turionifera, Spirodela intermedia*, and *Landoltia punctata*) (Ryman and Anderberg, 1999). *Landoltia punctata* is native to south-east Asia and Australia, but is an alien in parts of Europe (Hussner, 2012), and in the U.S.A. (Jacono, 2002). *Lemna valdiviana* is native in the Americas but has become invasive in parts of southern Europe (Iberite et al., 2011). *Lemna gibba* is native in Europe, Asia and North America (Hussner, 2012), but alien invasive in parts of southern Africa (Henderson, 2007). In Ireland, as well as much of Europe, *L. minuta* is an alien invasive species. *L. minuta* (Least Duckweed) is native to temperate regions of North and South America (Stace, 2010). This duckweed naturally occurs in a wide range of habitats, including mountainous regions, up to 4000 m of altitude, to temperate and tropical regions (Landolt, 1986). Invasive, alien *L. minuta* has been spreading in Europe for the last 40 years (Gassmann et al., 2006). It is widespread in Europe, including Germany (Hussner et al., 2010), Belgium (Halford et al., 2011), Poland (Wójciak and Urban, 2009), Hungary (Lukács et al., 2014), France (Jovet and Jovet-Ast, 1966), Italy (Conti et al., 2005), and Malta (Misfud, 2010). In England *L. minuta* is becoming more prevalent, since being discovered in 1977 (Bramley et al., 1995). *L. minuta* was first found in Ireland in Co. Cork in 1993. Since, it has been reported at 133 lowland sites and is now considered an established species (Lucey, 2003). Another invasive species (but not belonging to the Lemnaceae) that is frequently observed in the same aquatic habitat as *L. minuta* is the freshwater fern *Azolla filiculoides*. This species is originally from North and South America where it is widespread from Patagonia to Alaska, including the Caribbean Islands (Wagner, 1997). The species has been recorded in 19 European countries, and based on the perceived threat level, the European and Mediterranean Plant Protection Organization (EPPO) included it on the EPPO List of Invasive Alien Plants (Hussner, 2012). *A. filiculoides* was introduced in the British islands at the end of the nineteenth century as an ornamental plant (Simonsen, 1968), but it is currently widely spread across these islands (Preston and Jane, 2014). The water fern has been reported to cause severe problems by impeding navigation, water flow and angling and by causing fish kills and damage to wetland biodiversity (Janes, 1998).

*L. minuta* and *A. filiculoides* often co-occur with the native *L. minor* (Preston and Jane, 2014) and appear to compete for the same habitat (Dickinson and Miller, 1998; Ceschin et al., 2016). A comparative approach with native species has often been used in studies of invasive species (Daehler, 2003; Bossdorf et al., 2005; Funk, 2008). This approach consists of a comparison of alien and native species, and attempts to identify traits associated with invasiveness such as biomass allocation, growth rate, size and fitness. Comparative studies are particularly meaningful when comparing species that occupy the same ecological niche, and/or species that are closely related, as this facilitates the identification of differences that may be responsible for invasiveness (Mack, 1996). Similarly, a comparative analysis of invaded and non-invaded habitats can identify characteristics that determine the invisibility of habitats.

In this study, the abundance, growth-rate and distribution patterns of two alien freshwater plants (*Lemna minuta* and *Azolla filiculoides*) were compared with those of *L. minor*. Environmental parameters were compared between invaded and non-invaded habitats. Specifically, the hypothesis was tested that a combination of high growth rates and nutrient enrichment will facilitate invasion. The data will contribute to the understanding of colonization events, and ultimately inform aquatic management approaches.

## Materials and methods

This study is comprised of three parts.

### 1-presence and abundance of three species of floating macrophytes in natural ponds

#### Area investigated

**Figure 1:**
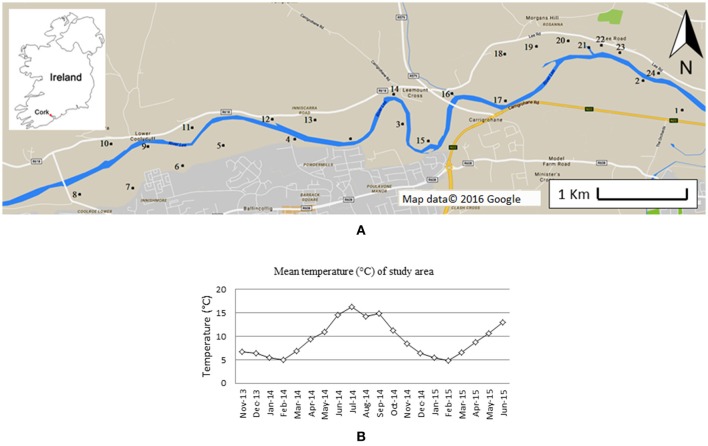
Map of the area monitored **(A)**. Numbers indicate the sites investigated. Insert shows the location within Ireland. In **(B)** the average monthly temperature during the time frame of this study is showed. Data were provided by the Irish Meteorological Service, and measured at Cork airport meteorological station (51°50′50″ N 8°29′10″ W). Figure adapted from Paolacci (2016).

The ponds investigated are situated along the north and south banks of the River Lee in south-west Ireland, 5 km west of Cork City (Figure 1A). The area includes a range of small, still- and slow-flowing water bodies. A total of 24 still ponds (indicated in Figure 1A, coordinates in Supplementary Table 1) were selected for further study. The ponds selected included water bodies with heterogeneous characteristics (e.g., different North-South aspect, canopy-cover, proximity to farms and/or houses). Most ponds are <100 m^2^, and the depth generally varies between 50 and 150 cm. The bedrock in the area is composed of Devonian sandstone, covered by Carboniferous limestone. The area is used for agricultural and recreational activities. In winter, the entire area is subjected to inundation. Occasionally, some of the water bodies dry out, completely, or partially in summer.

#### Monitoring approach

Field monitoring started in November 2013 and lasted till June 2015. The monthly average temperature during the period of field monitoring is shown in Figure 1B. A total of 6 observations were made of macrophyte abundance (November 2013, April, June and November 2014, May and June 2015), one of shading (in June 2014) and two of water nutrient content (April and June 2014).

The presence and abundance of three species of floating macrophytes was quantified for each of 24 still- and slow-flowing water bodies in the study area. A 50 × 50 cm floating quadrat was used to estimate the percent cover of each species in each quadrat. Four random throws of quadrats were carried out in each water body and the mean of the 4 quadrats was calculated. It was assumed that the percent cover of the quadrats reflected the percent cover of the water body. The values estimated were translated into the following classes:

–absent
–present (1–25% of the surface of the water body covered)
–abundant (26–75% of the surface of the water body covered)
–dominant (76–100% of the surface of the water body covered)

The canopy produced by other plants (trees, bushes and reeds growing around and inside the ponds) on the surface of the whole water body was visually estimated. Each site was classified according to the following scale:

Pond not shaded (0% canopy)

Pond partially shaded (up to 25% of canopy)

Pond mostly shaded (25–75% of canopy)

Pond completely shaded (75–100% of canopy)

Total Oxidized Nitrogen (TON) and Total Phosphorus (TP) concentrations were quantified in each water body in early spring (April 2014) and in early summer (June 2014). The content of TP in the water was determined using the ascorbic acid method (Murphy and Riley, 1962), while the TON content was measured using a DR 2800 Spectrophotometer following the cadmium reduction method (Koroleff, 1972).

### 2-growth-rates of three species of floating macrophytes in natural ponds

**Figure 2:**
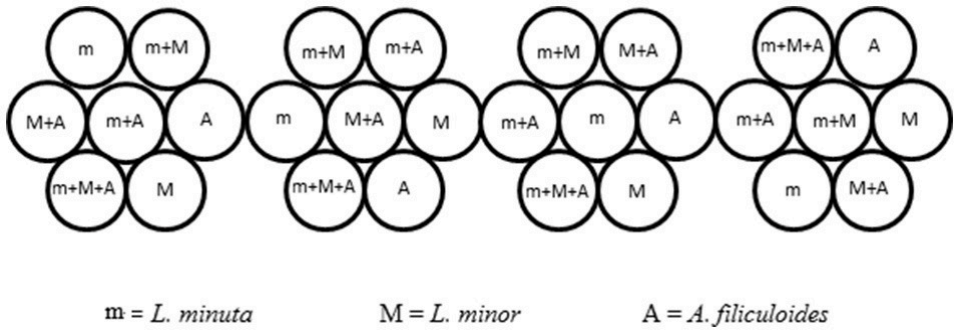
Design of the floating enclosures for the field experiment. Four composite enclosures (each comprised of 7 sub-units) where placed in each of the 9 ponds selected for the experiment. The 4-times replicated enclosures contained all the possible combinations of the 3 species. Figure adapted from Paolacci (2016).

Ponds were selected based on the most abundant free floating macrophyte, generating three categories of ponds. In three selected ponds *L. minuta* was more abundant than the other species investigated, while in a further three ponds *L. minor* was most abundant. In the final three ponds none of the three species was present. The experimental design would also have required the inclusion of three ponds in which *A. filiculoides* was the abundant species, but at the time of this experiment none of the sites presented this characteristic. In each of the selected ponds four plastic, floating enclosures were placed. Each enclosure was divided into 7 circular sub-units (short 12-cm lengths of plastic piping of 10.5 cm diameter, perpendicular to the water surface) inside which we grew all possible combinations of the three species (the three species alone, *L. minuta* with *L. minor, L. minuta* with *A. filiculoides, L. minor* with *A. filiculoides* and the three species all together) (Figure 2). The four enclosures were bound together and tied with a rope to the edge of the water body so that they had a certain degree of freedom, but they could not be dragged too far by the current or the wind. A net was placed over the enclosures to prevent birds from accessing the sub-units and to prevent leaves and other wind-blown material from accruing. Growth was quantified by placing biomass of each of the three species in the appropriate 10.5 cm diameter sub-unit (30 mg of *L. minuta*, 50 mg of *L. minor*, and 80 mg of *A. filiculoides* starting biomass). Plants were then allowed to grow for a period of 4 weeks. At the end of the 4 week period the plant material was collected, the different species were separated, weighted and the RGR was calculated according to the formula of Connolly and Wayne (1996):

$$\begin{array}{l} \text{RGR}=\text{ln }\left(\text{Yf}/\text{Yi}\right)/\text{t} \end{array}$$

Where Yi is the initial biomass or the initial number of fronds, Yf is the final biomass or final number of fronds, t is the time in days and ln is the natural logarithm.

The experiment started the 20th of May 2014 and it ended the 20th of June 2014. Over the 3 weeks of the experiment the average maximum temperature registered at the Cork meteorological station was 15°C, while the average minimum temperature was 8.6°C.

### 3-temporal changes in growth rate and abundance of three floating macrophytes in mesocosms

The mesocosm experiment started in November 2013 and finished in November 2014, and was designed to identify dynamic differences in abundance and growth rate across the different seasons. Twenty-eight mesocosms were constructed by sinking plastic containers 31 cm deep into the ground. Containers had a diameter of 50 cm. Mesocosms were filled with rain water after construction. No top-up was required for the duration of the experiments. In each mesocosm, a small amount of sediment (750 g) was added as a source of nutrients. This sediment was gathered from the same pond from which all the three macrophyte species were collected (pond 16 in Figure 1A). Following addition of sediment, mesocosms were left plant-free for 4 days in order to allow the sediment to settle, and for some of the nutrients contained in the sediment to dissolve in the water. When the experiment started the concentration of soluble orthophosphate (SRP) in the water was 0.03 ± 0.001 mg/l and the concentration of nitrate was 4.1 ± 0.3 mg/l. These concentrations were similar to those observed in several water bodies along the river Lee where the three macrophyte species are naturally present.

Mesocosms contained either single macrophyte species, combinations of two species, or a mixture of three species. All plant material used for the experiments was collected from a pond within the study area (pond number 16 in Figure 1A). In November 2013, a total of 5 cm^2^ of floating macrophytes, of all the possible combinations of the three species, was placed in the mesocosms. Each combination was replicated four times, with replicate mesocosms located at random within the experimental array, to avoid spatial confounding. The mesocosms were covered with wide-mesh netting to prevent birds interfering with the experiment. The relative area occupied by each species in each mesocosm was estimated every month with the method of the point intercept (Floyd and Anderson, 1987).

**Figure 3:**
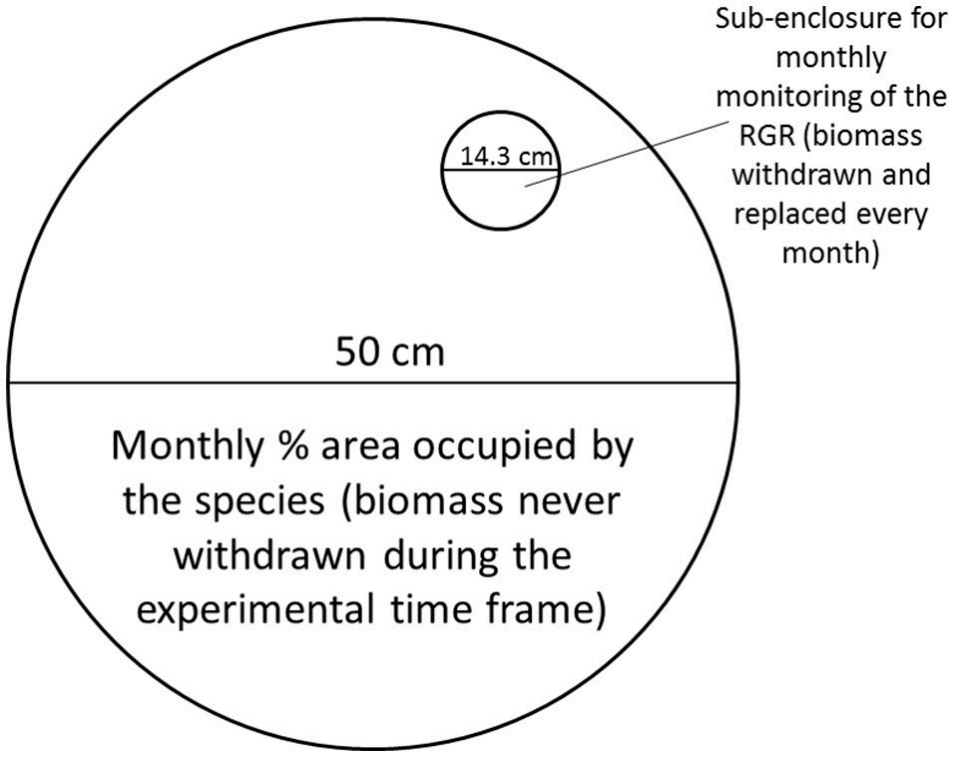
Design of the mesocosm. The main compartment was used to monitor standing stock, while the sub-enclosure was used to determine growth rates for the three species when grown in allopatric conditions. Figure adapted from Paolacci (2016).

Additionally, in each of the mesocosms containing only one species a small sub-enclosure with a diameter of 14.3 cm was placed in order to monitor growth rates every month (Figure 3). Every month 50 mg of the same species as present in the rest of the mesocosm was added to the sub-enclosure, and this was removed and weighed after 2 weeks. The RGR was calculated as detailed before. The monthly mean temperature for the experimental time frame can be observed in Figure 1B.

### 4-data analysis

All the statistic tests were performed using IBM SPSS Statistic 22.

### Field monitoring

The relation between the presence and/or abundance of the three macrophyte species and TON, TP and canopy cover was investigated by carrying out Kendall's Tau b test.

### Field experiment

A 2-way ANOVA was run in order to identify differences in RGR between the three species grown in allopatric conditions in the different categories of pond. For each of the three species, a 2-way ANOVA was run to analyse the differences in RGR for plants grown in different categories of ponds, and again to statistically compare growth in allopatric or sympatric conditions.

### Mesocosm experiment

A 2-way repeated measures ANOVA was used to analyse the differences in RGR and in percentage of surface cover between the three species, grown in allopatric conditions, in the different months of the year. For each of the three species, another 2-way repeated measures ANOVA was run in order to analyse the differences, in RGR or % of surface cover, between the species grown in allopatric or sympatric conditions. Sphericity was assessed with Mauchly's test. The degrees of freedom were corrected using Greenhouse-Geisser estimates of sphericity. When a statistically significant interaction was found, an analysis of simple main effects was performed by running a one-way repeated measures ANOVA for the different subsets of variables. Bonferroni-corrected *t*-tests were applied for pairwise comparisons.

## Results

### 1-monitoring the presence and abundance of three species of floating macrophytes in natural ponds

Of the three investigated species, *L. minor* and *L. minuta* were the most abundant in the 24 water bodies monitored during the 2 years of the study. *L. minor* was found in 12 water bodies, *L. minuta* in 11 water bodies and *A. filiculoides* only in three water bodies. In five water bodies, both *L. minuta and L. minor* were found, in three of them co-occurring at the same time, in the other two the two species were present at different times. In only one pond all three species were found together. In two other ponds *A. filiculoides* co-occurred with *L. minuta*. Thus, *A. filiculoides* never occurred in the absence of at least one *Lemna* species. No floating plants were observed in six of the 24 ponds (Table 1).

**Table 1:**
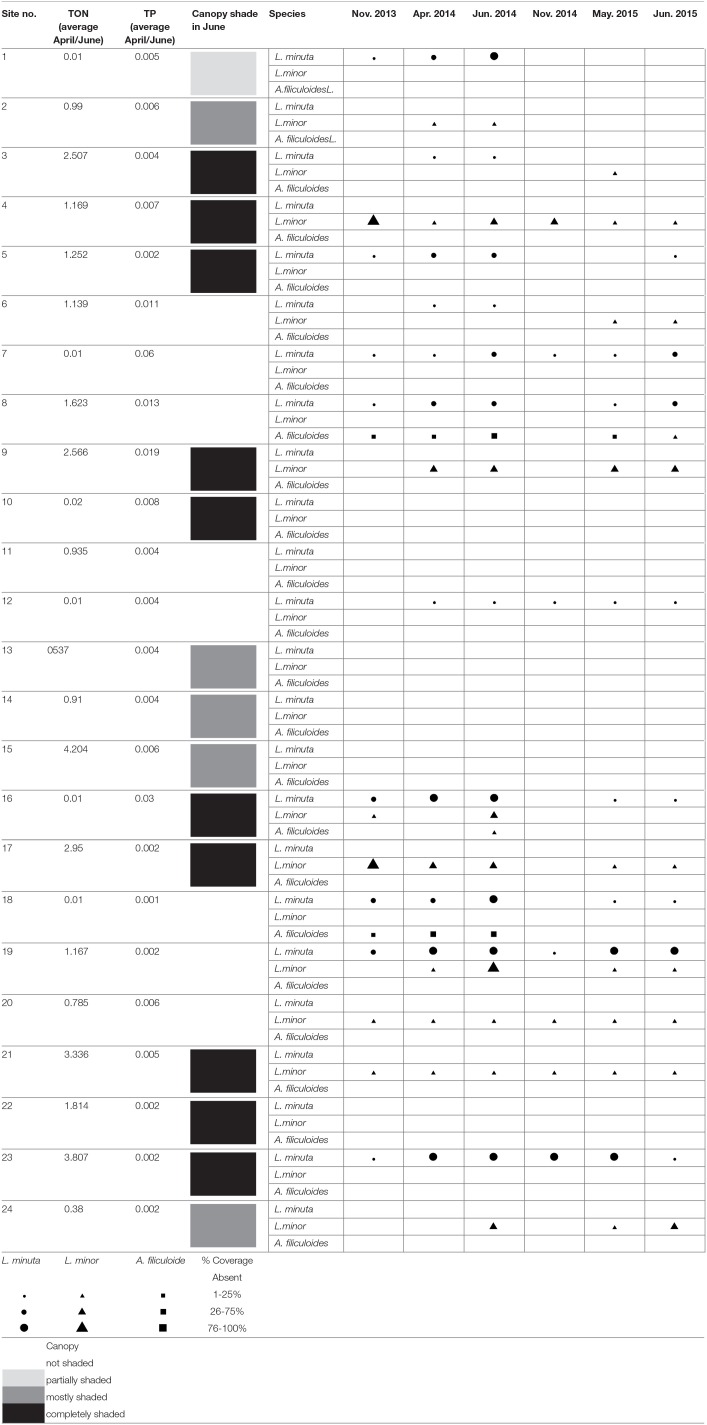
Presence and abundance of *L. minuta, L. minor*, and *A. filiculoides* in 24 ponds monitored at six different time points.

*TON, Total Oxidized Oxygen; TP, Total Phosphorus, and canopy cover are also shown*.

Analysis of water nutrient content revealed that Total Oxidized Nitrogen (TON) ranged between 0.01 and 5.207 mgl^−1^ (mean 1.798 mgl^−1^) across all 24 water bodies in April 2014 and between 0.01 and 3.807 mgl^−1^ (mean 0.88 mgl^−1^) in June 2014. Total Phosphorus (TP) ranged between 0.001 and 0.118 mgl^−1^ (mean 0.0132 mgl^−1^) across the 24 water bodies in April 2014 and between 0.001 and 0.06 mgl^−1^ (mean 0.0062 mgl^−1^) in June 2014.

There was no clear relationship between the percentage surface cover of the three species and TON and TP concentrations. Also the analysis of canopy cover did not reveal any significant correlation with the occurrence of the three species (Table 2).

**Table 2:** Correlation coefficient (Kendall's Tau *b*-test) between the percentage surface cover of *L. minuta, L. minor*, and *A. filiculoides* with Total Oxidized Nitrogen (TON), Total Phosphorus (TP) and Canopy cover.

	**Correlation coefficient**		
	***L. minta***	***L. minor***	***A. filiculoides***
TON	−0.193	0.044	−0.195
TP	−0.054	0.268	0.183
Canopy	−0.219	−0.174	−0.163

*Correlation refers to the data collected in April and June 2014 for TON and TP and June 2014 for canopy cover. The data of percentage coverage by aquatic macrophytes refers to the same months of 2014*.

In general, the three species were more widespread in the first year of monitoring. The comparison of June 2014 with June 2015 (months with the highest presence of the three species), showed that the number of ponds in which *L. minuta* was present decreased from 11 (in 2014) to 8 (in 2015). The 8 water bodies that contained *L. minuta* in 2015 also contained *L. minuta* in 2014. The number of sites in which *L. minor* was present decreased from 10 to 8. One of the ponds that contained *L. minor* in 2015 did not contain *L. minor* in 2014. The number of sites in which *A. filiculoides* was present decreased from 3 to 1.

### 2-growth rates of floating macrophytes in ponds with different macrophyte populations

**Figure 4:**
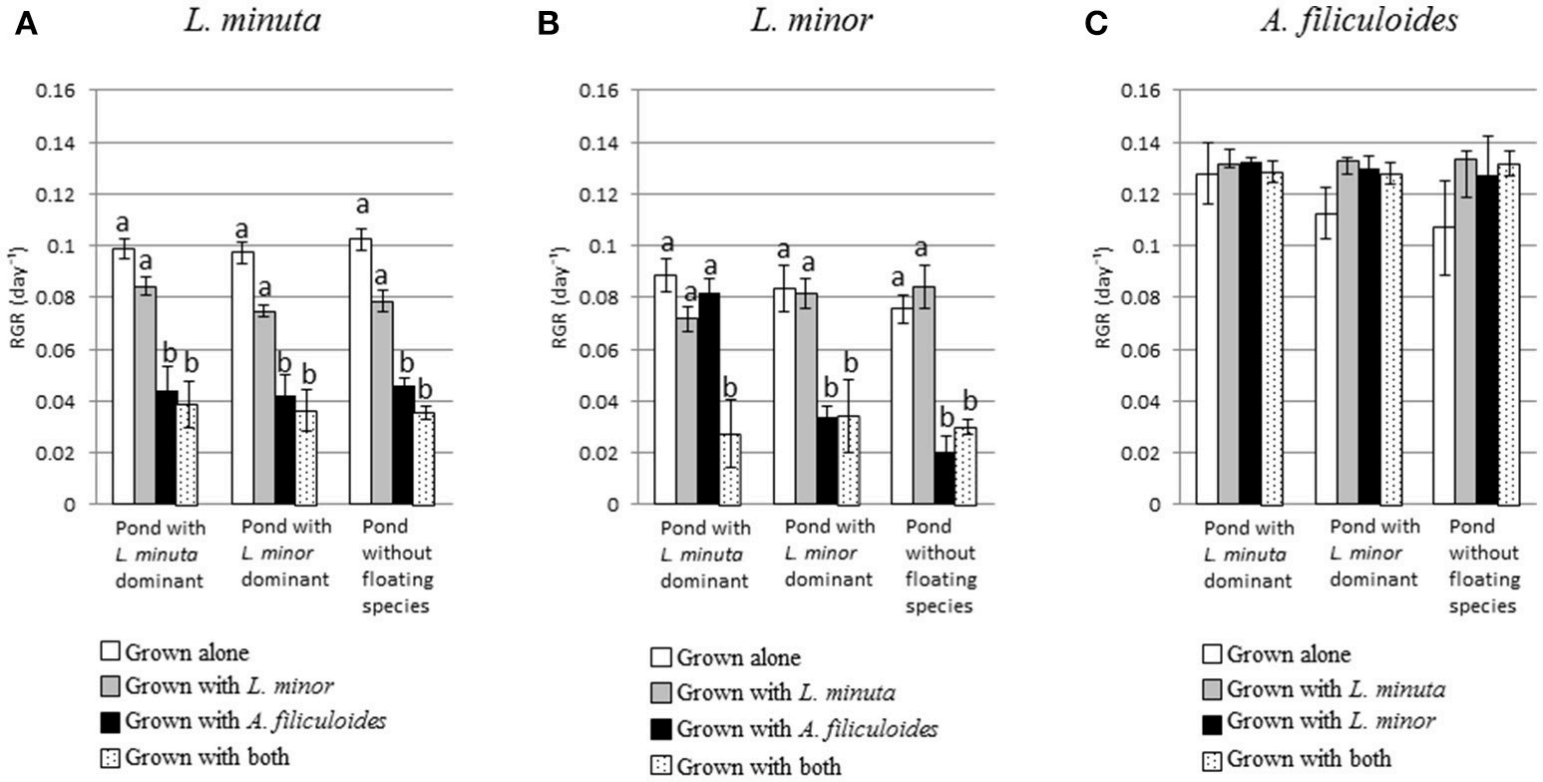
Mean (±1 S.E.) RGR of *L. minuta* **(A)**, *L. minor* **(B)**, and *A. filiculoides* **(C)** grown in different mixtures of species (alone, with one of the other two species or with both) in the three different categories of ponds (ponds dominated by *L. minuta*, ponds dominated by *L. minor* or ponds with floating species absent). Different letters indicate significant differences between species for each pond type. Figure adapted from Paolacci (2016).

Pond category (i.e., ponds naturally dominated by *L. minor*, or *L. minuta* or lacking macrophytes) did not affect the growth of the three species when these were introduced and raised in enclosures (Figures 4A–C). In fact, Table 3 shows that for none of the three species the interaction between category of pond and species mix was significant. Yet, when *L. minuta* and *L. minor* were grown in the presence of *A. filiculoides* the RGR of the Lemnaceae was significantly reduced (Figures 4A,B). The RGR of *L. minuta* was significantly affected by both other species, Tukey *post-hoc* tests revealed that RGR of *L. minuta* grown alone was significantly greater than when grown with *L. minor* (*p* = 0.002), with *A. filiculoides* (*p* < 0.001) and with both species (*p* < 0.001). Also for *L. minor* RGR was significantly affected by the presence of the other species. Tukey *post-hoc* tests revealed that RGR of *L. minor* grown alone was significantly greater than when grown with *A. filiculoides* (*p* = 0.003) and with both species (*p* < 0.001).

**Table 3:** Summary of 2-way ANOVAs for each species, of the effects of mix of species (alone, with one of the other two species and with both) and pond category (ponds dominated by *L. minuta*, ponds dominated by *L. minor* or ponds with floating species absent) on RGR.

**Source**	**Type III sum of squares**	***df***	**Mean square**	***F***	**Sig**.
***L. minuta***					
Mix	0.023	3	0.008	165.625	0.000
Pond	0.000	2	0.000	2.221	0.130
Mix * pond	0.000	6	8.161E-05	1.729	0.157
Error	0.001	24	4.719E-05		
Total	0.133	36			
Corrected Total	0.025	35			
***L. minor***					
Mix	0.023	3	0.008	137.944	0.000
Pond	0.000	2	7.059E-05	1.244	0.306
Mix * pond	0.000	6	2.874E-05	0.507	0.797
Error	0.001	24	5.674E-05		
Total	0.179	36			
Corrected Total	0.025	35			
***A. filiculoides***					
Mix	0.001	3	0.000	3.579	0.029
Pond	0.000	2	8.412E-05	0.727	0.493
Mix ^*^ pond	0.001	6	0.000	0.867	0.533
Error	0.003	24	0.000		
Total	0.587	36			
Corrected Total	0.005	35			

### 3-growth of three macrophyte species grown in allopatric conditions in mesocosms, over a 12 month period

**Figure 5:**
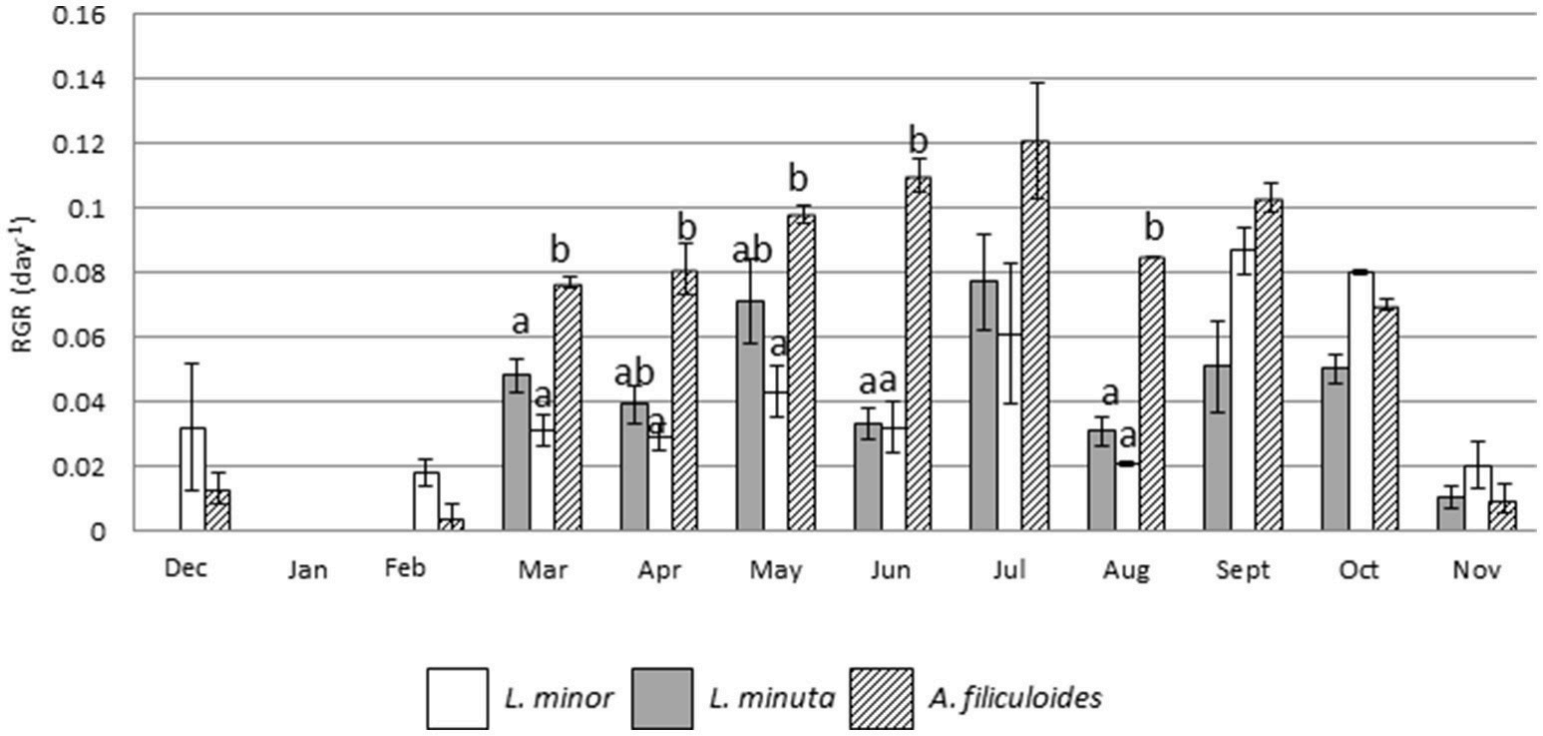
Mean (±1 S.E.) RGR of *L. minuta, L. minor*, and *A. filiculoides*, grown in sub-enclosures in outdoor mesocosms from December 2013 to November 2014. Different letters indicate significant differences for each month.

Outdoor mesocosms were constructed to facilitate the study of growth throughout the four seasons. The analysis of growth under allopatric conditions showed that the three species had a reduced RGR in the colder months (from November to January), while their RGR increased from spring onwards. Growth of *L. minuta, L. minor*, and *A. filiculoides* peaked in the summer period between May and September. For *L. minuta* the highest RGR was 0.077 ± 0.015 day^−1^ in July. For *A. filiculoides* the highest RGR (0.12 ± 0.02 day^−1^) was obtained in July. For *L. minor* RGR peaked in September (0.087 ± 0.007 day^−1^). Lowest growth rates were measured in January, when none of the three species grew. Both in December and February, only *L. minor* and *A. filiculoides* displayed growth, while *L. minuta* only displayed substantial growth from March (Figure 5).

Analysis using 2-way repeated measures ANOVA highlighted that there was a significant difference in RGR, both between species and between months. The interaction between the two factors was also significant (Table 4). In the colder months (from December from February) *A. filiculoides* did not significantly outgrow the other two species, but in March the RGR was higher than for *L. minuta* (*p* = 0.042) and *L. minor* (*p* = 0.02). The water fern continued to grow faster than *L. minuta* and *L. minor* in the following months until September (although not always significantly, see Figure 5).

**Table 4:** Results of 2-way repeated measures ANOVA.

	**Source**	**Type III sum of squares**	***df***	**Mean square**	***F***	**Sig**.
Species	Greenhouse-Geisser	0.030	1.282	0.023	22.444	0.009
Time	Greenhouse-Geisser	0.116	1.584	0.074	35.030	0.002
Species * time	Greenhouse-Geisser	0.036	2.290	0.016	6.868	0.021

*Interaction between species (L. minuta, L. minor, and A. filiculoides) and time on RGR of the three species, grown in allopatric conditions in mesocosms*.

### 4-surface cover of the three macrophyte species grown in different species mixtures in mesocosms

**Figure 6:**
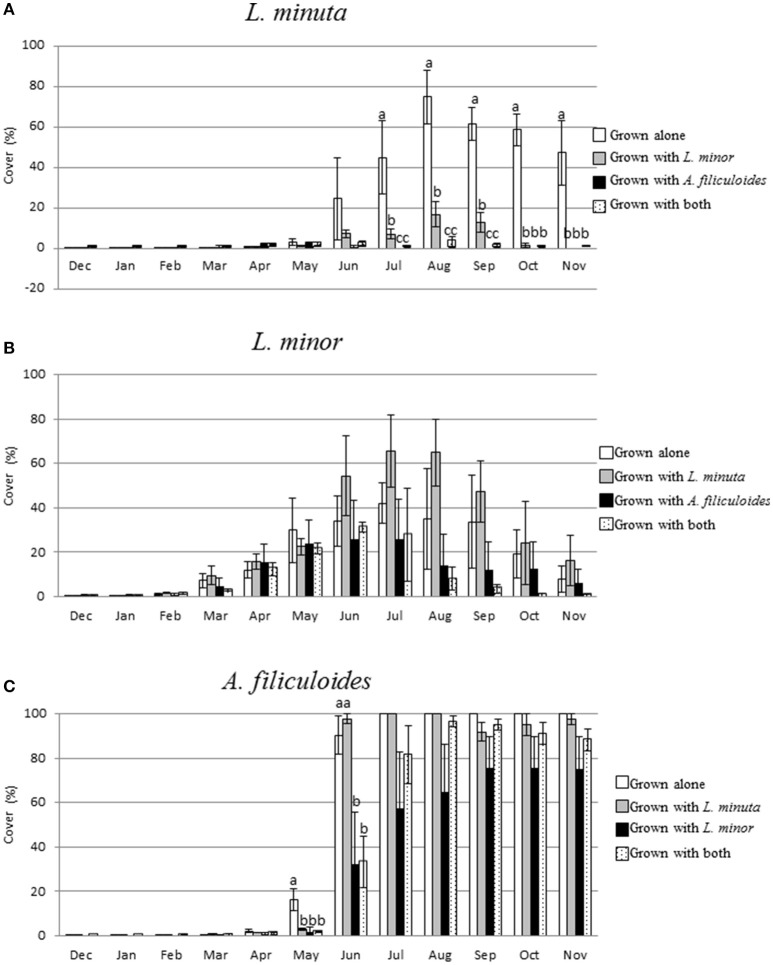
Mean (±1 S.E.) Percent surface cover of *L. minuta* **(A)**, *L. minor* **(B)**, and *A. filiculoides* **(C)**, grown in different mixtures (alone, with one of the other two species or with both), in outdoor mesocosms from December 2013 through to November 2014. Different letters indicate significant differences between species in each month. The total surface area (100%) was 1,753 cm^2^. Figure adapted from Paolacci (2016).

The percentage surface cover was measured every month for each of the three species grown in different mixtures. When grown alone, in the period December through to February, none of the species covered more than 2% of the surface area. From March to May only *L. minor* increased its percentage of surface cover. *A. filiculoides* increased its percentage of coverage only from May onwards, and *L. minuta* only from June. In the period July to November *A. filiculoides* covered up to the 100% of the mesocosm surface. The highest percent cover reached by *L. minuta* was 74.9 ± 13.4% in August. For *L. minor*, the highest percentage of cover was 42.64 ± 9.17% in July (Figure 6).

A 2-way repeated measures ANOVA showed that there was a significant difference in surface cover between species and months. The interaction between the two factors was also significant (Table 5). From November to March 2015 there was no significant difference in surface cover between the three species, while, in April, *L. minor* surface cover was significantly higher than that by both *L. minuta* (*p* = 0.024) and *A. filiculoides* (*p* = 0.046). From June to November *A. filiculoides* always covered an area significantly greater than both *L. minuta* and *L. minor*. In these summer months, *L. minuta* covered an area greater than *L. minor*, but only in October was this difference significant (*p* = 0.006).

**Table 5:** Results of the 2-way repeated measures ANOVA.

**Source**		**Type III sum of squares**	***df***	**Mean square**	***F***	**Sig**.
Species	Greenhouse-Geisser	28118.260	1.069	26312.858	13.462	0.031
Time	Greenhouse-Geisser	115234.953	1.355	85037.560	55.056	0.001
Species * time	Greenhouse-Geisser	39591.563	1.700	23286.138	9.429	0.021

*Interaction between L. minuta, L. minor, and A. filiculoides and time in the comparison of the surface cover by the three species grown in allopatric conditions*.

The monthly surface cover by each species grown alone was compared with the cover of that same species grown with one of the other two species or with both. Data analysis by 2-way repeated measures ANOVA was separately run for each species. Surface cover by floating macrophytes strongly depended on the season. For all the 3 species there was a significant interaction between species mixture and time (Table 6).

**Table 6:** Results of a 2-way repeated measures ANOVA.

**Source**		**Type III sum of squares**	***df***	**Mean square**	***F***	**Sig**.
***L. minuta***						
Mix	Greenhouse-Geisser	21169.910	1.231	17201.353	69.012	0.001
Time	Greenhouse-Geisser	11512.566	1.439	7999.795	17.413	0.010
Mix * time	Greenhouse-Geisser	24376.574	1.472	16560.542	10.882	0.022
***L. minor***						
Mix	Greenhouse-Geisser	9772.164	1.715	5697.963	1.346	0.331
Time	Greenhouse-Geisser	33933.470	1.414	24001.549	13.787	0.016
Mix * time	Greenhouse-Geisser	12316.381	1.868	6594.991	1.722	0.260
***A. filiculoides***						
Mix	Greenhouse-Geisser	10883.911	1.174	9269.019	5.053	0.096
Time	Greenhouse-Geisser	351738.347	1.603	219371.285	215.791	0.000
Mix * time	Greenhouse-Geisser	16870.522	2.185	7722.444	2.485	0.156

*Interactions between different species mixtures and time in the comparison of the surface cover of the three species*.

The surface area occupied by *L. minuta* was significantly affected by both the species mixture and the month of the year (Table 6). Surface cover was strongly reduced when this species was cultured in the presence of the other two species, but only from July to November (Figure 6A). In July, *L. minuta* surface cover was reduced when grown together with *L. minor* and surface cover was nearly completely suppressed when *A. filiculoides* was present in the culture mix. A similar situation was observed also in the following months.

The surface area occupied by *L. minor* was also significantly affected by the month of the year, while the effect of the species mixture was not statistically significant (Table 6). The percentage surface cover of this species was reduced by the presence of *A. filiculoides*, in the period from July to November, but not significantly. The presence of *L. m inuta* had no effect on *L. minor* surface area (Figure 6B).

The surface area occupied by *A. filiculoides* was significantly affected by both the species mixture and the month of the year (Table 6). The most evident difference between species mixtures occurred in May and June, when *A. filiculoides* surface cover was reduced by the presence of the other species. From July to November there was a smaller difference between the surface coverage for this species grown alone and in sympatric conditions (Figure 6C).

## Discussion

The invasiveness of species and the invasibility of habitats are considered the key complementary parameters that determine the potential success of biological invasions (Alpert et al., 2000). Factors that increase habitat invasibility include high resource availability, limited competition with species present, ecological disturbances and the absence of environmental stressors (Alpert et al., 2000). Factors that increase the invasiveness of a species include rapid population growth, ability to deal with stressors and/or disturbances, and rapid dispersal. While invasiveness and invasibility have been investigated in depth separately, there is a gap in the literature for studies analyzing their antagonistic and/or synergistic effects on species distribution. This study integrates analysis of invasiveness and invisibility by measuring simultaneously growth rates, distribution and occurrence of two invasive (*L. minuta* and *A. filiculoides*) and a native species (*L. minor*) as well as resource availability and seasonality across a series of natural ponds and mesocosms.

### Distribution of *L. minor, L. minuta*, and *A. filiculoides* is not associated with resource availability

Surface cover was quantified for three free floating macrophytes across 24 natural ponds, which are all located in a wetland along the river Lee in south-west Ireland. Despite the apparent similarity of the ponds, as well as the proximity to one another, Lemnaceae (i.e., *L. minor* and/or *L. minuta*) were found in 18 ponds, but not in another 6 ponds, in the 2014 survey. Native *L. minor* was the most common species in ponds within the study area (12 out of 24), closely followed by the alien *L. minuta* (11 out of 24). The other alien, *A. filiculoides* occurred in only 3 out of 24 ponds, and can thus be considered rare in the study area. Interestingly, the 2015 survey showed that some ponds that contained *L. minor* or *L. minuta* in 2014, lacked these species in 2015 (e.g., ponds 1 and 2). Other ponds that did not contain *L. minor* in 2014, did so in 2015 (e.g., pond 3 and 6). Thus, it can be concluded that the distribution of the three species of free floating macrophytes is heterogeneous in both space and time. Spotty distribution of Lemnaceae has been reported previously. McLay (1974) reported heterogeneous distribution of *L. perpusilla*, and that the presence of the species was negatively associated with exposure to wind and waves, and positively linked with the presence of *Potamogeton* and *Scirpus*. Similarly, Kline and McCune (1987) reported on the heterogeneous distribution of *Wolffia columbiana* and *Wollfia punctata* across a series of small potholes clustered together in a small area in Montana, USA. As reported in this study, a heterogeneous distribution of Lemnaceae was found, notwithstanding distances of <50 m between potholes. In the study by Kline and McCune (1987) it was concluded that the heterogeneous distribution of Lemnaceae reflected environmental parameters. Although the heterogeneous distribution in space is, thus, a more commonly reported phenomenon, the current study adds an extra layer of complexity by demonstrating a heterogeneous distribution in time.

In general, distribution of a species depends on the match of an array of physico-chemical parameters with the specific environmental requirements of the species (Santamaría, 2002). For example, a study by Peeters et al. (2016) revealed the relative importance of phosphorus for growth and competitiveness of free-floating macrophytes, and the presence of many species of aquatic macrophytes is associated with eutrophic conditions (Carbiener et al., 1990). The study presented here revealed a substantial gradient of light and nutrients across the 24 natural ponds in the study area. However, the data presented in this paper show no association of nutrient or light levels with distribution suggesting that physicochemical conditions in ponds fulfill minimal requirements for the three species. In support, previous experimental work has shown that both *L. minor* and *L. minuta* grow well on a broad range of nutrient conditions (Paolacci et al., 2016). Furthermore, on a global scale, aquatic species tend to be more widespread than closely related terrestrial species, and distribution patterns are typically less affected by environmental factors (Santamaría, 2002).

The explanation that all ponds fulfill the minimal requirements for growth of all three macrophyte species, triggers the challenging question why 6 out of 24 ponds have no free floating macrophyte cover in 2014. This is a particularly intriguing question as some of these ponds contained substantial amounts of floating macrophytes in 2015. Using reciprocal transplanting experiments (Figure 4), it was demonstrated that all species are able to grow in all ponds, even ponds were the species do not naturally occur. It might have been anticipated that actual growth rates (RGR) depend on light and nutrient levels. However, this was not the case. Indeed, in complex natural environments, effects of nutrients or light can be masked by other environmental factors, leading to the lack of correlation between resource availability, presence and growth rate. This is consistent with work by Makkay et al. (2008) who reported that in many cases single physical or chemical variables fail to explain the variation in aquatic plant community composition. A detailed study of the replacement of *L. minor* by *L. minuta* in central Italy, also led to the conclusion that environmental factors cannot explain the outcompeting of *L. minor* by *L. minuta* (Ceschin et al., 2016). Thus, based on the data presented in this paper it is concluded that distribution of *L. minor, L. minuta*, and *A. filiculoides* is not limited by the prevailing physicochemical characteristics of the studied water bodies during the summer period, and in the experimental area.

### A high RGR is not associated with high abundance and/or wide distribution

Analysis of growth rates revealed that all species can grow in all ponds tested. Highest growth rates (RGR) in the field were noted for *A. filiculoides*. This species also had a dramatic negative effect on the RGR of the two species of Lemnaceae, when cultured together in close proximity. Analysis of the areas covered by the three macrophytes in the mesocosms further highlighted that *A. filiculoides* was able to reduce the coverage of *L. minor* and *L. minuta*. In contrast, in the absence of *A. filiculoides* both *L. minuta* and *L. minor* can cover a substantial part of the available surface (Figure 6). *A. filiculoides* appeared to suppress the growth of the two *Lemna* species, possibly due to its higher growth rate as argued by Filizadeh (2002), or possibly due to its morphological features that facilitate it taking over the space available. The importance of morphology was recognized by Clatworthy and Harper (1962), who observed that the floating macrophyte *Salvinia minima* can outcompete *Spirodela polyrhiza* through physically overtopping the latter. Irrespective of the underlying mechanism of competition, we found that a relatively rare species (i.e., *A. filiculoides*) displays the highest RGR, the highest surface cover and is the most competitive, in the studied ponds, during the summer. Conversely, *L. minor* has the widest distribution in the ponds studied (Table 1), but displays the lowest RGR values (Figure 4) in monocultures under field conditions. In a way these data are surprising, as many, but not all, studies have associated high Relative Growth Rates (RGR) with competitiveness (Grotkopp and Rejmánek, 2007; Dawson et al., 2011). While “competitors” typically display a high potential RGR, stress adapted plants are characterized by a more modest RGR (Grime and Hunt, 1975). Interestingly, Santamaría (2002) has characterized aquatic habitats as inherently stressful, implying that high RGR values are not necessarily relevant to explain growth and competitiveness. Consistently, based on the data presented in this paper, it is concluded that the RGR, even when measured in a natural habitat, is not necessarily a good indicator for either abundance or distribution.

### Differences in the phenology of three free floating macrophytes

The period from late spring to early autumn, is the main period of growth for free floating aquatic macrophytes in Ireland (Figure 5). To explore in more depth the importance of seasonal growth, a mesocosm experiment was used to monitor RGR throughout the seasons. In general, RGR values tend to be highest in summer, and lowest in winter (Figure 5) patterns that are consistent with both lower temperatures and light-doses in the latter period. Percent surface cover was also lowest in winter (Figure 6). However, there are significant distinctions between the three species studied. *L. minor* displayed a significantly higher RGR than the other two species in winter (December–February) (Figure 5). *L. minor* also displayed significant surface cover in March and April, well ahead of the two other species (Figure 6). Phenological variations, whereby a species exploits resources at a time that other species are not active, can play an important role in competitive relationships (Regehr and Bazzaz, 1976). An “early start” can give a species a competitive advantage relative to a fast growing competitor that “arrives” later in the growing season. The relative ability of *L. minor* to grow in winter has been noted before. Reddy and DeBusk (1985) showed that the growth of *L. minor* is less influenced by seasonal changes than that of the water fern *A. caroliniana*. Also, Paolacci et al., (submitted) showed that *L. minor* can grow under lower temperatures than *L. minuta*, under laboratory conditions. Thus, we conclude that the three species of free floating macrophytes have different phenological cycles, with *L. minor* being able to maintain a low growth rate throughout much of the winter period in Ireland, and this may confer a competitive advantage.

### What determines the heterogeneous distribution of three free floating macrophyte species across the study area?

A remarkable finding of this study has been that the species with the lowest summer RGR, i.e., *L. minor*, is most widely distributed throughout the study area. In contrast, the species with the fastest summer growth, i.e., *A. filiculoides*, is relatively rare. Furthermore, this study has revealed a heterogeneity in time, with variations in the distribution of floating aquatic plants across ponds, between subsequent years. These two findings trigger the question what determines the distribution and abundance of the three species of free floating macrophytes in the studied system of ponds. Here we identify two important elements that determine this heterogeneous distribution.

1. Dispersal and heterogeneous distribution

Shifts in the community composition of floating plants have been observed in response to seasonal environmental factors such as flooding, drought and extreme temperatures (Bornette and Puijalon, 2011; O'Farrell et al., 2011). Ponds in the area investigated are subject to flooding in winter, at which all ponds become connected and subject to substantial currents that may wash away free floating macrophytes. Ponds may also be subject to drought in summer, at which stage there may be no surviving free floating macrophytes. In this study it was concluded that all studied species can grow during the summer in all ponds. Therefore, it can be argued that re-colonization of these ponds after winter flooding and/or summer drought is a determinant of vegetation composition and that dispersal pathways need to be considered when analyzing vegetation dynamics. A study by Nishihiro et al. (2014) linked heterogeneous distribution of floating-leaved *Trapa japonica* to limitations in seed dispersal. Conversely, the “spotty” distribution of floating plants (Wolek, 1983; Kline and McCune, 1987) has sometimes been attributed to chance dispersal. However, bird mediated dispersal may facilitate targeted distribution in the waterfowl rich study area. Bird mediated dispersal has been well described for short distance dispersal (Jacobs, 1947; Reynolds et al., 2015), especially for species of Lemnaceae (Coughlan et al., 2015a,b). Thus, the possibility that Lemnaceae distribution patterns in the field study area reflect dispersal patterns needs to be considered.

(2) Phenological factors

In this study it was found that native *L. minor* displays stronger growth during the winter months than *L. minuta* and *A. filiculoides* (Figures 5, 6). Potentially this can give *L. minor* a competitive advantage over the other species. Thus, it can be envisaged that a long winter and/or cool spring will benefit *L. minor*, and result in increased abundance. Conversely, a warm spring and/or hot summer might favor the other two species. Such a scenario is in agreement with work by Dickinson and Miller (1998), who showed that the floating aquatic macrophyte *Salvinia minima* was highly competitive during the summer, negatively affecting cover by both *Azolla caroliniana* and *Spirodela punctata*. However, competitive superiority of *S. minima* was found to be seasonal, with summer gains being reversed due to a relative intolerance of winter conditions. Similarly, Peeters et al. (2013) observed that milder winters are correlated with a relatively higher abundance of free-floating plants and, as a result of shading, a reduced presence of submerged plants. Thus, the possibility that *L. minor* prevalence in the field study area reflects winter growth needs to be considered.

## Conclusion

*A. filiculoides* displays the highest RGR in this study, and exerted a negative influence on growth rates and surface cover of *L. minor* and *L. minuta*. Despite such apparent invasiveness, *A. filiculoides* was relatively rare in the study area. Rather, the species most present throughout the study area was *L. minor* which has the lowest RGR under field conditions in summer. Therefore, this study proves, for the first time, that the invasiveness of the species during the summer months is not necessarily reflected in the actual distribution pattern in natural water bodies. In fact, the alien species *L. minuta* and *A. filiculoides* are under-represented in the monitored area. It is concluded that the interaction of several factors, including growth under winter-conditions and/or dispersal after disturbances, is the major determinant of the abundance and distribution of *L. minor, L. minuta* and *A. filiculoides* in the study area. These results can have implications in the management of invasive species, suggesting that an integrated analysis of invasiveness and invasibility is necessary to decide whether an intervention is required or not.

## Author contributions

SP designed and carried out the experiment, analyzed and interpreted the results and wrote the paper. SH supervised the design and execution of experiments, the data analysis, the interpretation and the manuscript. MJ supervised the design and execution of experiments, the interpretation of results and the manuscript.

### Conflict of interest statement

The authors declare that the research was conducted in the absence of any commercial or financial relationships that could be construed as a potential conflict of interest.
