# Supplementary material for: Competition Between Lemna minuta, Lemna minor, and Azolla filiculoides. Growing Fast or Being Steadfast?
Source: Front Chem. 2018 Jun 14;6:207. doi: 10.3389/fchem.2018.00207 (PMC6010541; doi:10.3389/fchem.2018.00207)
Supplement: Supplementary file 1 [file Table_1.DOCX]

| Waterbody | Latitude | Longitude |
| --- | --- | --- |
| 1 | 51.892843 | -8.531734 |
| 2 | 51.895062 | -8.525105 |
| 3 | 51.895225 | -8.574605 |
| 4 | 51.893905 | -8.589299 |
| 5 | 51.893072 | -8.599597 |
| 6 | 51.89229 | -8.605667 |
| 7 | 51.891575 | -8.612367 |
| 8 | 51.891741 | -8.614858 |
| 9 | 51.893527 | -8.610699 |
| 10 | 51.893897 | -8.615992 |
| 11 | 51.894662 | -8.603649 |
| 12 | 51.895326 | -8.59256 |
| 13 | 51.895898 | -8.583327 |
| 14 | 51.897965 | -8.576325 |
| 15 | 51.893601 | -8.571032 |
| 16 | 51.897624 | -8.568047 |
| 17 | 51.897519 | -8.559594 |
| 18 | 51.899935 | -8.559874 |
| 19 | 51.901459 | -8.557579 |
| 20 | 51.90226 | -8.552388 |
| 21 | 51.901587 | -8.548277 |
| 22 | 51.901591 | -8.546123 |
| 23 | 51.901102 | -8.543642 |
| 24 | 51.896009 | -8.52175 |

**Supplementary table 1.** Coordinates of the waterbodies monitored.
